# Factors associated with ownership and utilization of insecticide treated nets among children under five years in sub-Saharan Africa

**DOI:** 10.1186/s12889-022-13347-x

**Published:** 2022-05-10

**Authors:** Kennedy Diema Konlan, Nathaniel Kossi Vivor, Isaac Gegefe, Linda Hayford

**Affiliations:** 1 Department of Public Health Nursing, School of Nursing and Midwifery, University of Health and Allied Sciences, Ho, Ghana; 2 College of Nursing, Yonsei University, 50-1, Yonsei-ro, Seodaemun-gu, Seoul, 03722 Korea; 3 Public Health Unit, Municipal Hospital, Ho, Ghana; 4 General Nursing Unit, St. Anthony’s Hospital, Dzodze, Ghana

**Keywords:** Insecticide Treated Bed Net, Malaria, Prevention, Under Five Children, Treatment, Utilisation

## Abstract

**Background:**

Insecticide-treated net (ITN) is a cost-effective means to control malaria and morbidity in under-five children. This study synthesizes the factors associated with using the ITN as a malaria prevention tool in sub-Saharan Africa.

**Methods:**

There was an advanced search of four electronic databases, including PubMed Central, CINAHL, EMBASE, and Google Scholar, and identified articles between 2016 to April 2021. Following the title, abstract and full-text reading, 13 articles were deemed appropriate for this review. All the researchers developed, discussed, and accepted a matrix to extract relevant information from the studies. A convergent synthesis was adopted and allowed for integrating qualitative, quantitative, and mixed-method studies and transforming them into qualitative findings.

**Results:**

Household and caregiver related factors that influenced utilization of the ITN were, Household heads having two or more sleeping places, a knowledge that ITN prevents malaria, the presence of hanging ITNs, high literacy, living female-headed households, birth spacing, unmarried mothers, and antenatal clinic attendance promoted utilization. Perceived malaria risk was a critical determinant of ITN ownership and utilization. Some factors that hindered the use of the ITN included hotness of the weather, absence of visible mosquitoes, cost, inadequate number, rooms designs, unaffordability, insufficient knowledge on causes of malaria, and poor attitude to use. Specific ITN factors that hindered use were color, chemicals use, odor, and shape.

**Conclusion:**

It is important to use integrated multi-sectoral and culturally appropriate interventions to encourage households to prioritize and utilize the ITN in under-5 children.

## Background

The World Health Organization (WHO) estimated that malaria is a significant health burden, and nearly 3.3 billion people in 97 countries (located mainly in the tropics) are at risk of malaria (1–3). Malaria has a psychological, social, and demographic burden on inflicted persons and is responsible for a chunk of deaths and increasing healthcare costs (4, 5). Malaria diverts time away from income-generating activities, which leads to reduced household income, especially among the poor. This highlights the additional economic and social repercussions of the disease (4, 5). The WHO indicated that in 2015, enumerated malaria cases were about 214 million, and 438,000 malaria deaths (3). In Africa, the impact is devastating and causes 78% of all deaths in under-5, i.e., children less than five years old (3). Under-5 deaths serve as a good indicator of the nature and status of countries' health care utilization and access. The Roll Back Malaria (RBM) Partnership's Monitoring and Evaluation Reference Group recommends under-5 child mortality as the primary indicator of malaria control impact (1, 3, 6, 7).

There have been increasing efforts to curb the spread of malaria, especially in Sub-Saharan Africa. Insecticide-treated net (ITN) is one of the cost-effective interventions for the prevention and control of malaria (8, 9). Other interventions like indoor residual spraying, destruction of mosquito breeding sites, prompt and adequate treatment of infected cases are also instructive (2, 4, 8, 9). The WHO and the RBM campaigners have been critical in recommending that where ITN are unavailable for the entire household, priority is to under-5 and pregnant women (2). This effort has led to the free distribution of ITNs in the antenatal clinics for pregnant women and the child welfare clinics for under-5 to promote access and increase utilization. An Increase in donor funding for malaria prevention interventions in the previous decade also had a corresponding increase in the use of ITN, especially among under-5 (7).

In most countries, the most significant reduction in Under-5 mortality was attributable to ITN use, with a population attributable risk percentage of 11% (7). Also, UNICEF estimated that under-5 mortality in Africa declined by 20% between 2000 and 2010 (from 159 to 127 deaths per 1000 live births), with over one million children's lives saved due to malaria interventions (1, 10). Insecticide-treated bed nets (ITNs) or long-lasting insecticidal nets (LLIN) have been a cornerstone of malaria control for decades (2). Several studies have identified the factors associated with ITN distribution (11) and usage (6, 12), especially among under-five children. These systematic reviews were conducted when the Millennium Development Goals (MDGs) were in full implementation. The MGDs, especially goal seven, focused on combating HIV/AIDS, malaria, and other diseases. This heightened interest in preventing malaria by targeting vulnerable populations especially under-5 in malaria-endemic areas of sub-Saharan Africa (13). Also, several interventions mainly targeted those factors that influence use among vulnerable populations by eliminating related barriers, improving awareness, and reducing cultural bias (6, 12). The MDGs were evaluated to achieve the fundamental goal related to malaria prevention (6). However, the malaria menace continues to be a significant challenge among vulnerable populations living in sub–Saharan Africa. These might be influenced by varied factors that probably were not considered during the period of implementation of the MDGs. Several factors are noted to have influenced the utilization of the ITN among children under-5 (13), but those studies that identified this factor are mainly sporadic and generally uncoordinated (14).

Also, the factors that influence the use of ITN among vulnerable populations are relatively fluid and multidimensional, and synthesis of these factors is imperative (13, 14). As these interventions remain instructive, child mortality remains a bane to countries with malaria incidence, and related complications are still estimated to be a significant contributor (15). Studies conducted to help improve the ownership and utilization of ITN are widespread and sporadic in Sub-Saharan Africa, with no specific collation of the factors that influence ownership and utilisations among under-5. This study synthesized the factors associated with ownership and utilization of the ITN among children under-5 in sub-Saharan Africa.

## Methods

### Literature Search

The Preferred Reporting Items for Systematic Reviews and Meta-Analyses (PRISMA) framework guided the identification, screening, and selection of studies for this review (16–18). There was a detailed search using the advanced search option of four electronic databases, i.e. (PubMed central, Cumulative Index to Nursing and Allied Health Literature—CINAHL, EMBASE, and Google Scholar), and identified articles between 2016 through to April 2021. The Population Intervention Comparison Outcome (PICO) criteria were used in creating the search parameters. Children under-5 were the population, the intervention included factors associated with the use of ITN, there was no comparison, and the outcomes were ownership and utilization of the ITN. The Keywords and the associated terms used as either MeSH terms (for medical terms), free text with synonyms incorporating the appropriate Boolean operators for the literature search included ("Factors associated" OR "factors influencing") AND (Child* OR "under five" OR preschool OR "0 to 5 years") AND ("Insecticide-treated bed net" OR "bed net").

### Article selection

**Fig. 1:**
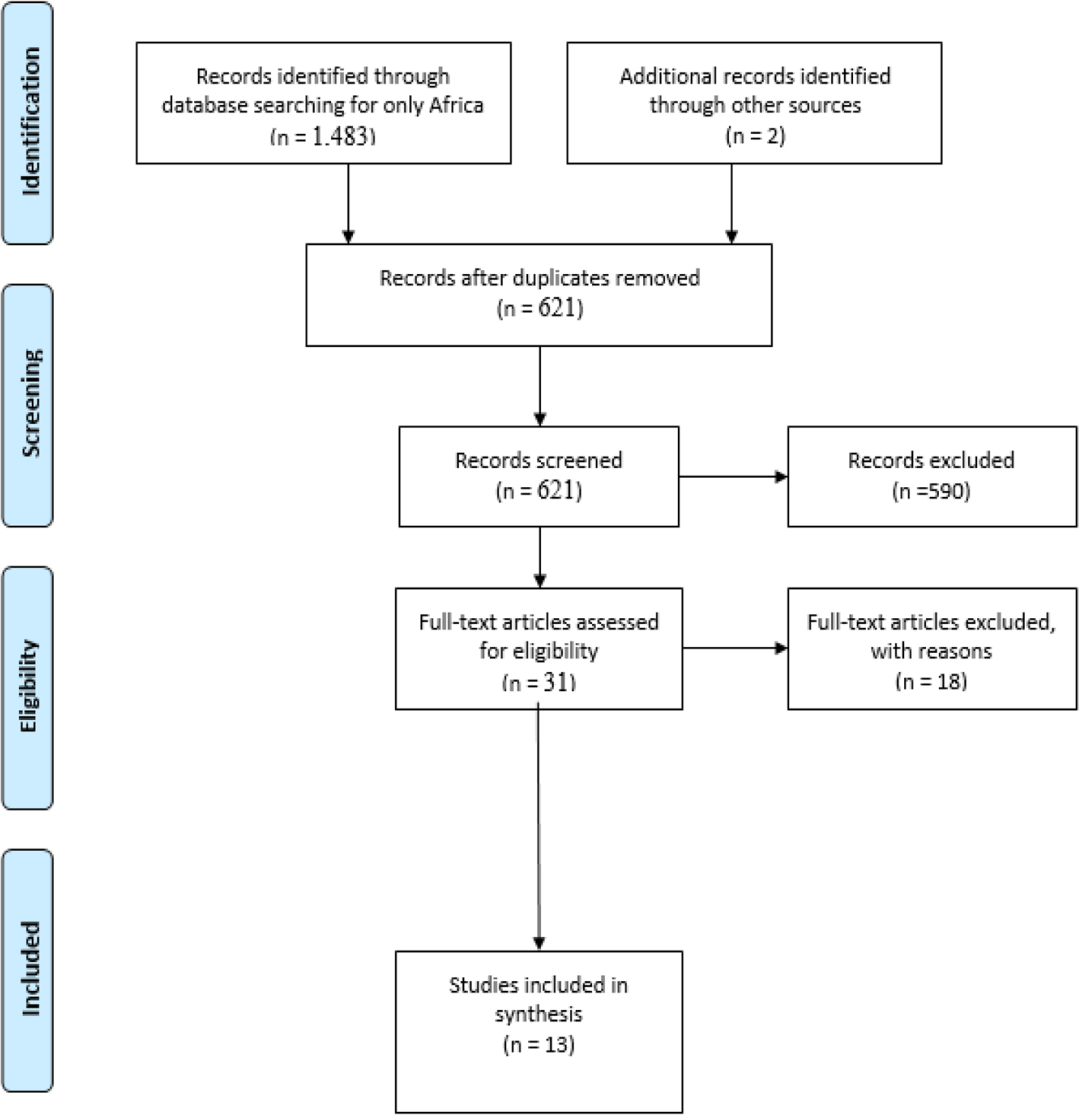
PRISMA flow chart

In the search, using the MeSH terms and the keywords incorporating the appropriate Boolean operators, 4,623 titles were retrieved and filtered from 2016 through to April 2021 to 2,272 titles. Those from Sub–Saharan Africa were 1,485 (1,483 titles from database search and 2 from other references). The Sub-Saharan Africa-specific studies identified were from PubMed (381), CINAHL (5), Embase (17), and Google Scholar (1,080). The identified articles were transported to Endnote X9 software, and duplicates were identified and removed to produce 621 titles. All the 621 titles were screened for appropriateness, and 381 titles were selected for abstract reading based on the titles relating to ITN use among under-5. After reading the abstract only, 31 abstracts were selected for full-text reading (identified only articles that expressly indicated the factors associated with the ownership and utilization of the ITN among under-5). Upon full-text reading, 13 articles were selected as appropriate for this review. The details of the article selection process are shown in the PRISMA diagram (Fig. 1). The reasons for exclusion of articles during the full-text reading included 1) the study conducted among the general population and not children under-5 years, 2) evaluated and implemented program by an organization on malaria prevention including indoor residual spraying, 3) assessed factors associated with the distribution of the ITN in antenatal clinics and among pregnant women, 4) described the impact of malaria on under-5 mortality and morbidity including the trends of infections and complications associated with malaria, and 5) identified the factors associated with malaria infection.

### Exclusion criteria

This study did not include any study with the under listed characteristics: assessed knowledge on ITN use; described infection and/or knowledge on malaria; assessed knowledge on ITN use in the general population; identified malaria infection among children under -5; described the factors associated with under-5 child mortalities and indicated indoor residual spraying as malaria prevention measures; targeted school-going age children or those above five years.

### Quality appraisal

Using the Mixed Methods Appraisal Tool (MMAT) version 2018, two researchers independently assessed the quality of all the included studies (19). Where there were discrepancies between the two assessors, the discussion was revisited another time after each assess read the manuscript once more. These repeated discussions continued until a consensus was reached. Where an agreement could not be reached after multiple attempts, a third researcher was consulted, and a majority decision prevailed. The MMAT contains methodological quality criteria for appraising qualitative, quantitative, and mixed methods studies. The MMAT for quantitative and qualitative sections evaluates the appropriateness of the study aim, study design, methodology, recruitment of participants, data collection, analysis of data, presentation of results, discussions, and conclusions. The researchers did not assign the overall quality score, which is discouraged by Hong et al. (19).

### Data extraction and synthesis

All the researchers developed, discussed, and accepted a matrix to extract relevant information from the studies. The extracted information were authors, study design, settings, population, analysis, factors that promote ownership and utilization, and those that hinder utilization. Two of the researchers independently extracted data from each study, and the results were compared; where there were discrepancies, it was resolved through consensus.

A thematic data analysis approach was used to synthesize essential information from articles. Study codes generated upon thorough reading of the extracted data collated into subthemes, and similar subthemes merged to form themes. Descriptive themes were assessed to create meaning beyond the original data, leading to new, interpretive analytical themes. A convergent synthesis design was adopted, and this allowed for the integration of results from qualitative, quantitative, and mixed-method studies and transformed them into qualitative findings (20).

## Results

The search within the four databases identified 1,485 titles and 621 after duplicate removal for Sub-Saharan Africa from 2016 to April 2021. After reviewing abstracts, only 31 abstracts were identified for the full-text screen, and 13 articles were eligible. These articles were selected because they were English-based articles that assessed the factors associated with using the ITN for under-5.

### Characteristics of the studies

The studies were primarily conducted in sub–Saharan Africa. The specific locations and countries of each study were Mirab-Abaya District, Gamo-Gofa zone (21, 22), and Kola Diba Town, North Gondar Amhara region (23) in Ethiopia; Malawi (24); Madagascar (25); Bamenda health district in Cameroon (26); Zambezia province in Mozambique (27); Budondo sub-county (28), Mbarara municipality (29) in Uganda (30); Osun state, in the southwest (31), Nigeria (32); and in Rwanda (33). The adopted sampling methods include systematic (22, 23) and simple random sampling (28, 33). The other sampling methods were cluster (24–26, 30, 32), stratified (24, 27), four-stage (31), three-stage sampling (29), and multistage stratified sampling (21).

The studies used the quantitative approach except for a mixed-methods study (31). The data collections tools were questionnaires, home observations (28)), focus group discussions (31), and other measured parasitemia (29). Data analysis methods adopted included the descriptive (22, 23, 25, 26, 30, 31), univariable and multivariable logistic regression (21, 22, 24, 27, 28, 32, 33). The other analysis methods were multivariable regression analysis (24). The studies were funded by the Cameroon Field Epidemiology training program Department of Microbiology and Parasitology (26), Nigeria Field Epidemiology Training Programme (31), and the President's Malaria Initiative through the United States Agency for International Development through MEASURE Evaluation cooperative agreement (32).

### Thematic results

The key findings were organized into integrated and presented in specific themes. The main themes that emerged were prevalence of ownership and utilization of ITN; household and caregiver related factors that influence utilization of ITN in under-5 children; caregiver knowledge that influences utilization of the ITN; and barriers to ITN use in under -5 children. These key factors are presented in Table 1. Table 1 shows the prevalence of ownership and utilization of the ITN; determinants of ownership and utilization of the ITN; and key findings that are subsequently integrated into the thematic analysis.

**Table 1:** Distribution of key findings

Reference	Prevalence of Ownership and Utilisation	Determinants of Ownership and Utilisation	Key Findings
Admasie (2018) [22]	• Access to ITN (91.0%) • Used previous night (37.2%) • Observed ITN mounted (42.9%)	• Maternal and/or caregivers' age • Family size and sleeping space • Inadequate knowledge on the proper use of ITN	• Factors that influence ITN in under-5 were age of caretakers 31–44 years (AOR = 0.03, 95% CI 0.01–0.07), maternal age ≥ 45 years (AOR = 0.05, 95% CI 0.01–0.58), family size ≤ 5 members, (AOR = 11.23, 95% CI 4.31–29.24) and sleeping space ≥ 2, (AOR = 13.59, 95% CI 4.40– 41) • Used ITNs for unintended purposes like raping over mattresses to protect from bugs, for grain and fruit carrying, to spread grains for the sunshine, room curtains, and fishing
Alemu (2018) [23]	• Accessed ITN through mass distribution (99.2%) • Used ITN the previous night (91.9%, CI = 88.5–95%) • prioritize children in ITN (91.9%)	• Perceived malaria risk of household head • Houses built from cement • Knowledge of the treatment of ITN	• Household heads determine the use of ITN (59.2%) • Houses made from cement are 97.7% times less odd to use ITN • People who do not know about malaria transmission are 90.8% times less likely to use ITN • Some respondents (93.1%) did not treat the ITN
Finlay (2017) [25]	• Mean ITN ownership was 2 per household • Received campaign ITN (70.1%) • Utilisation (84.2%)	• Mass media and pre-campaign messages • Receiving post-campaign visits	• Only 70.1% reported receiving two or more campaign LLINs during the 2019 distribution, as 13% could not have access • There were critical weaknesses in the household registration and voucher distribution process, including charging "hidden fees"
Fokam (2017) [26]	• ITN ownership (63.5%) • Slept under ITN the previous night (47.2%)	• Gender, age, and educational level of the household head • ITN density in households • Weather suitability of ITN like heat, suffocation • ITN related factors like torn	• There was an association between ITN use and gender, educational levels of household head, environmental factors, and ITN density • The primary sources of ITN were the free mass distribution (87.7%), antenatal clinic (7%), gifts (3.3%), and purchases (2.1%)
Moon (2016) [27]	• Households' ownership of ITN (64.3%) • Under-5 sleep under ITN in 2010 (50%) and in 2014 (60%)	• Households headed by females and or having higher education • Household size • having electricity, and • more considerable household monthly income, • Travel time to a health facility	• Pregnant (58.6%) reported sleeping under ITN the previous night in 2010 compared to 68.4% in 2014 • As travel to health facility increases (1 h), 13% lower odds of sleeping under the ITN (OR 0.87: 95% CI 0.74–1.01, *p* = 0.07) • Factors that influence the use of ITN in under-5 children were household size, female-headed households, and having electricity
Moscibrodzki (2018) [28]	• Households' ownership of at least one ITN (40%) ●ITN available to only (27%) of household members	• awareness of ITN benefit • having under-5 child, • Household size • ITN obtained free	• The odds of an ITN correctly used (i.e., to sleep under) after adjusting for potential confounders were significantly lower for those obtained free • Factors that influence utilization were awareness ITN benefit (OR = 1.72, 95% CI 1.11–2.68, *p* = 0.02), having under-5 (OR = 1.11 CI 0.99–1.25, *p* = 0.07), household size (OR = 1.05 CI 1..-1.20, *p* = 0.05) • The odds of ITN being correctly used was significantly lower for those obtained free (OR = 0.33, 95% CI 0.21–0.51, p,0.01)
Nkoka (2019) [24]	• ITN use increase from 57.8% (95% CI 56.1–59.4) in 2010 to 69.0% (95% CI 67.4- 70.5) in 2015	• The educational level of the mother • Residing in a female-headed household • lack of ITN supply	• There was increased ITN usage among under-5 in the five years • Residing in a female-headed household living in homes that had poor ITN supply significantly reduced odds of ITN usage
Gonahasa (2018) [30]	• ITN ownership (65.0%) • Slept under ITN the previous night (39.5%)	• Time since the last campaign • Household wealth • Presence of under-5 • Age and gender of household heads	• In Uganda, ITN ownership, coverage, and use were all well below desired targets 2.5–4.5 years after the ITN distribution through a national campaign • Household wealth and time since the last campaign were the strongest predictors of household ITN ownership
Israel (2018) [31]	• Ownership of at least one ITN (82.9%) • Under-5 slept under ITN the previous night (58.6%)	• Formal education of caregivers • knowledge about ITN • Net color, size • Free distribution	• The significant sources of information on ITN were mass media (94.2%) • ITN protects against other insects' bites (99.8%) • Some believe the free ITN is more effective than the ones purchased • Some indicated ITN only comes in white color and gets dirty quickly
Simpson Nuwamanya (2018) [29]	• Owned ITN after free distribution (98.8%) • Use ITN for under-5 (91.9%)	• Gender of household head • ITN size, shape, worn-out, perceived poor quality, and allergic reaction to chemicals • Insufficient sleeping space • Environmental conditions • Knowledge of household heads	• Ownership of LLIN was very high, and Parasitaemia among the under-5 was very low • No childhood malaria episodes reported in the home in the last 12 months (OR = 1.69, 95% CI 1.02–2.83) were all associated with ITN use • Persons who did not sleep under the ITN the previous night indicated lack of sufficient space to hang, perceived poor quality, not having enough ITN
Ruyange (2016) [33]	• Households owned at least one ITN (72.2%)	• Household size • Employed and or educated mothers • Children born to a married mother or mother living with a partner, • Born to a mother who had 1–4 or more than four ANC visits	• Risk factors related to ITN non-use at the individual, household, and community levels include poverty, education, birth spacing, and antenatal clinic attendance • Protective risk factors for ITN use included households with more than three ITN (0.39 [0.33–0.47]), mothers who attended 1–4 to antenatal clinics during pregnancy (0.45 [0.29–0.69]), more than four antenatal clinic visits during pregnancy (0.39 [0.21–0.70]), mothers married or living with a partner (0.43 [0.36–0.52]), educated mothers (0.77 [0.65–0.91]), and households in higher community wealth quintile (0.71 [0.59–0.84])
Tassew (2017) [21]	• Ownership of at least one ITN (89.9%) • use ITN the previous night (85.1%)	• Two or more sleeping places • knowledge that ITN prevents malaria • Presence of a hanging ITN • Walls of the house plastered or painted > 12monts ago	• Poor knowledge of the transmission and the symptoms of malaria and vector control measures to prevent malaria were associated with ITN use • ITN were found to be out of use or in poor repair (30%)
Zalisk (2019) [32]	• Use of ITN were 40.5% in the highest wealth quartile 69.9% in the lowest wealth quartile (*p* < 0.0001)	• Adequate number of ITN • Caregivers exposed to malaria information • Residence • Wealthy quartiles	• Caregiver exposure to ITN-related malaria messages improves the use of ITNs in under-5 • significant associations were not found between ITN use and sex or age of the child, caregivers' educational attainment or household ownership of a television

### Prevalence of ownership and utilization of ITN

ITN ownership was 40% (28) and 82.9% (31), while availability was 27% of all household members (28) as shown in Table 1. Among households with at least one under-5, 91.0% had one ITNs (22). ITNs were obtained during antenatal care or child immunization visits (32) as 70.1% were received during the 2019 distribution campaign, with 13% lacking access (25). The prevalence of ITN use in under-5 was varied as 37.2% (22), 47.2% (26), 50.0% (27), 58.6% (31), 91.9% (23) used ITN a night before the survey in Table 1. There was an increased ITN usage among children under-5 within five years (24), while Uganda recorded a decrease in usage (30).

### Household and caregiver related factors that influence utilization of ITN

Factors such as having an under-5 child (28), female gender (26, 28), the literacy level of the household head (26), weather conditions (26), ITN density (26), and pregnancy status (28) were significantly associated with possession and usage in under-5. Households with less or equal to five members were more likely to have an under-5 sleep under ITNs (22, 27), while others indicated there was no impact of household size on ITN utilization (26). Household heads having two or more sleeping places (21), being female (24), having formal education (24, 27, 31, 33), unmarried mothers (24), attending antenatal clinic (27, 33), and adequate birth spacing (33) were significant factors that promoted ITN utilization in under-5. Another factor influencing ITN utilization was having a hanging place within the household sleeping area and having the room walls painted more than twelve months ago (21). Factors associated with the use of ITN in under- 5 for female-headed households included having electricity (27) and larger household monthly income (27). Another factor that promoted ITN use was the free distribution of ITN (22, 29, 33). Other factors that positively influence use were ITNs obtained during antenatal care or child immunization (32). Reasons for ITN non-use at the individual, household, and community level include poverty (33), the time elapsed since the last campaign, presence of an under-5, age and gender, and relationship to household heads (30). It was also noted that during free distribution and owning a radio was associated with higher LLIN access (25).

### Caregivers' knowledge influenced utilization of ITN in under-5

Perceived malaria risk and awareness of the benefits of ITN were essential determinants of ownership and utilization (23–25, 28, 31). Poor knowledge of the transmission mechanisms, symptoms of malaria, and vector control measures adversely influenced the use of ITN (21). The odds of ITN use correctly (i.e., to sleep under) after adjusting for potential confounders were significantly lower for those obtained free (28, 32). Caregivers' inadequate knowledge of the causes of malaria (22) and the lack of knowledge that ITN prevents malaria (21) significantly influenced the use in under-5. Caregiver exposure to ITN- related malaria messages (32, 33) and social behavior change communication messages (32) improved the use of ITNs among under-5. Also, Awareness creation made through the mass media increased the likelihood of using the ITN for under-5 (22). A high literacy of ITN was associated with increased use (25). Significant sources of information on ITN use were radio, television (25, 31), and healthcare workers (31). Mass distribution of LLIN led to its utilization (29, 31). A moderate proportion of ITN was out of the use or in poor repair (21).

### Barriers to ITN use in under-5

Some factors that hindered the utilization of the ITN included hot weather (22, 26, 31), absence of visible mosquitoes (22), cost, and unaffordability (22, 31). Other factors were the ITN inappropriate to room shape and size (22), poor attitude to use (18) and lack of ownership (22, 25), negligence, suffocation, and seasonal use (22). The specific ITN related factors that hindered utilization were ITN color (31), shape and quantity (22), unpleasant odor (41.3%) (31), and reaction to chemicals (75.5%) (31). Other factors include time since the last campaign, the number of residents in the household and sleeping space, and the socio-economic status of household heads (30, 33). As travel time to health facilities increased, utilization of the ITN reduced (27, 30). The ITN related factors that hinder utilization included torn and worn-out nets (26). Others indicated the ITN was washed and not hanged, expired, or complained of seasonal weather variations that made it difficult to use (26). Others indicated the ITN was not beneficial (26). Most households used the ITNs for unintended purposes or inappropriately, like wrapping over mattresses to protect from bugs, carrying grain and fruit, and fishing (22).

## Discussion

Malaria is endemic in the poorest areas of Africa, predominantly the African sub of the Sahara. This scoping review identified 13 studies that described the factors associated with the use of the ITN in under-5 children in Sub-Saharan Africa. Several factors were synthesized, and the main themes that emerged were prevalence of ownership and utilization, household and caregiver factors that influence utilization, caregiver knowledge, and barriers to ITN utilization among under-5. This demonstrates the varied factors that influence the utilization of this key malaria prevention tool among vulnerable populations. The studies were geographically limited to a few countries, including Ethiopia, Nigeria, Malawi, and Rwanda, and were published from 2016 to 2021. This emphasizes the need to tailor research to identify factors that influence the use of the ITN as a malaria prevention tool as cultural, socio-economic, and policy discrepancies in each country. Identifying this intricacy is instructive in the fight. Many of the studies identified the socio-demographic, policy, economic, and cultural factors associated with the use and ownership of the ITN, especially among families with under-5. These factors interact in a way that continues to hinder the effective use of the ITN in these vulnerable populations (8, 9, 34). The interaction of these factors was similar in Asia, where the predictors for participation in two malaria interventions programs were assessed (35). It is increasingly imperative to eliminate malaria, including severe anemia in children. Hence, governments, malaria control authorities, health care providers, and community leaders must institute measures that tackle and address socio-demographic factors responsible for the increasing spread of the disease (7). Some of these typical demographic factors include income levels, formal education, knowledge level, occupation, number of people within a household and in a living area, and elements that border on the empowerment of community members.

The availability and utilization of the ITN in the households ranged from a minimum of 40.0% (28) to 82.8% (31). This demonstrates the countrywide and geographical discrepancies in the ownership and utilization of the ITN within Sub-Saharan Africa. The differences in the average availability of the ITN for the use of under-5 children further emphasize the need for policymakers to identify and specifically target households for interventions including education (to increase awareness and knowledge), and distribution of the ITN, as free distribution was determined to be associated with increased utilization (36). Knowledge of malaria transmission, ITN handling, and malaria treatment were correlated to utilization among under-5. Increasingly tailored-based campaigns were identified as influential in instituting and allowing an under -5 use ITN. Improving maternal literacy, family income levels, and empowerment of household heads was shown to depict a positive relationship for the likelihood of ownership and utilization of the ITN in children under -5. These views were emphasized in Asia for malaria controls in endemic communities (35). It is also worthwhile to note that the higher prevalence of ITN in families with at least an under-5 child could be attributed to the role of governments in ensuring the distribution of the ITN in ANC and post-natal clinics where under-5 children are targets.

One crucial factor associated with the endemicity of malaria was the lack of and incongruent knowledge on the use of the ITN. Some studies indicated improper use of the ITN, where some people used it for gardening with limited knowledge of use. This is identified in other studies where knowledge was seen as a significant barrier to malaria prevention (37). It was shown that knowledge levels were a significant barrier to access, utilization, and appropriate use of the ITN within the household. Also, the need to prioritize the vulnerable populations in instances where there is limited availability of the ITN for the entire household was only noted in a few studies. Knowledge issues have been a barrier identified in other systematic reviews of malaria prevention (7, 35).

The factors that influence the ownership and utilization of the ITN among under-5 children are multiple and diverse. These factors range from individual household factors to systematic societal challenges associated with the net. Household and caregiver related factors that influenced utilization of the ITN were household heads having two or more sleeping places, a caregiver’s knowledge that ITN prevents malaria, the presence of hanging ITNs within the sleeping area, high literacy of ITN among household members and caregivers, having living female-headed households, wide birth spacing among children, a child belonging to unmarried mothers, and the mother having antenatal clinic attendance. Also, perceived malaria risk was a critical determinant of ITN ownership and utilization. Some factors that hindered the use of the ITN included hotness of the weather, absence of visible mosquitoes, cost and unaffordability, inadequate number of ITN, incongruent rooms design, insufficient knowledge of causes of malaria and poor attitude to use. Specific ITN factors that hindered use were color, chemicals, odor, and shape.

## Strengths and limitations

This scoping review provides timely evidence from the literature on the factors associated with the ownership and utilization of the ITN among under-5 children. In the region, research appeared sporadic and uncoordinated, with an increasing need to have one-unit scholarly work that integrated all the factors associated with using the ITN. Understanding these essential influencing and predicting factors to ownership and utilization of the ITN in Sub-Saharan Africa will aid in developing and implementing policies that can influence the fight towards the elimination of malaria. There are several limitations associated with this review. There may be some data sublimations as this study was more open in integrating all the various elements of the predictive factors for the usefulness of the ITN in children under-5. Also, other relevant publications might have been excluded because of the narrow scope of search and exclusion of non-English-based articles. This study particularly identified and integrated only the factors associated with the ownership and utilization of the ITN based on their clinical significance and did not lend credence to the level of statistical significance associated with each predicting factor.

## Conclusion

Several factors that range from demographic, socio-economic, and knowledge gaps were identified as significant barriers to the access and utilization of the ITN among under-5. This creates the need to use integrated multi-sectoral and multi-foci approaches in identifying and addressing issues related to access and utilization of the ITN, especially with vulnerable populations. Also, the findings of this study call for using integrated approaches in research by assessing the various foci of the problems associated with ownership and utilization of the ITN within vulnerable populations as the malaria endemicity remains a keen challenge for eliminating and reducing under -5 mortality and morbidity. The use of culturally efficient means of distribution and encouraging use of the ITN for this specific population will serve as a panacea towards controlling malaria.

## Data Availability

Data from which the conclusions are based have been included in this manuscript, and no data is deposited in any data repository.
